# Conducting rapid research to aid the design of a health systems governance intervention in the Somali Region of Ethiopia

**DOI:** 10.3389/fsoc.2022.947970

**Published:** 2022-09-08

**Authors:** Pieternella Pieterse

**Affiliations:** School of Nursing, Psychotherapy and Community Health, Dublin City University, Dublin, Ireland

**Keywords:** health systems governance, citizen engagement, citizen audit, woreda-level budget planning and budget execution, Somali Region, Ethiopia, rapid research

## Abstract

**Introduction:**

The rapid research described in this chapter was conducted as an assignment for a UN agency in Ethiopia's Somali Region. The agency's aim was support the implementation of an interim citizen engagement intervention, with a view of supporting of the Ethiopian Government's Citizen Score Card at primary healthcare facilities and hospitals in future. Many health facilities in Somali Region struggle with budget shortages related to ineffective budget planning and budget execution at woreda health office levels. In this context, an intervention to first improve budget accountability, through the implementation of citizen audits, was proposed.

**Methodology:**

The rapid study focused on five woredas (districts) within Somali Region, where interviews were conducted with the heads of woreda health offices. In the same five woredas, directors of healthcare facilities were interviewed and offices and healthcare facilities were observed. The framework of assessment and analysis was based on health systems literature on fragile and conflict affected states guided the questions for the health authorities and health facility management.

**Findings:**

The research yielded five distinct *mini case studies* covering woreda health office planning and budgeting capacity and support (or lack thereof), and related impressions of challenges regarding healthcare delivery at health facilities in the same five woredas.

**Results:**

The findings demonstrated that the capacity for healthcare planning and budgeting Somali Region at woreda level varied significantly and that little guidance was available from regional level health authorities. Frontline health services clearly suffered from budget shortages as a result.

**Conclusion:**

The research provided an evidence base for the delay of the roll-out of the Community Scorecard implementation across Somali Region. In a context whereby health facilities remain under-resourced due to budgeting constraints, a citizen-service provider-focused accountability intervention would have been of limited utility. The rapid case study research, conducted by condensing the usual case study research process, allowed for the production of evidence that was “robust enough” to demonstrate heterogeneity and challenges regarding budgeting quality across the five research sites. This evidence clearly transcended the hitherto anecdotal evidence that woreda-level health budget planning remains an area that faces significant shortcomings.

## Introduction

In 2018, the Lancet Global Commission on High Quality Health Systems in the Sustainable Development Goals-era, published its report on the quality of healthcare in “Low- and Middle-Income Countries” (LMICs). It highlighted that over 8 million people in LMICs die every year from conditions that should be treatable by the health system (Kruk et al., 2018). The report's authors noted that

“In 2015 alone, these deaths resulted in US$6 trillion in economic losses. Poor-quality care is now a bigger barrier to reducing mortality than insufficient access. 60% of deaths from conditions amenable to health care are due to poor-quality care, whereas the remaining deaths result from non-utilization of the health system (Kruk et al., 2018, p. e1197).”

Ethiopia is a low-income country and despite impressive gains in that past two decades, in terms of the establishment of additional health facilities and the creation of greater access to healthcare opportunities, significant bottlenecks continue to exist (Assefa et al., 2018). Low vaccination rates, a lack of citizen engagement and low levels of trust in healthcare providers pervade, especially in regions of the country where pastoralist communities make up the majority of the population (Ethiopian Public Health Institute, Federal Ministry of Health, The DHS Program and ICF, 2019). The Lancet Commission report by Kruk et al. (2018) advocates a range of strategies to improve the quality of care in LMICs. In addition to important technical and clinical suggestions, its fourth “broad recommendation” focuses on improving accountability in the health sector:

“…Governments and civil society should ignite demand for quality in the population to empower people to hold systems accountable and actively seek high-quality care (2018, p. e1198).”

Interventions that promote accountability in public services in LMICs have been gaining in popularity since the early 2000s. Social accountability interventions have been used to create dialogue between citizens and service providers through the establishment of scoring instruments in which citizens rate services, or actual “citizen - service providers” meetings in which grievances can be aired (e.g., Molyneux et al., 2012; Joshi, 2017). Such programmes have predominantly been implemented or facilitated by external actors, such as Non-Governmental Organization (NGOs) or Civil Society Organization (CSOs) (O'Meally, 2013; Holland and Schatz, 2016), however, national governments have also started to use social accountability tools to institutionalize citizen engagement for the purpose of promoting greater accountability (e.g., Feruglio and Nisbett, 2018).

In Ethiopia, a significant citizen engagement intervention, the Ethiopia Social Accountability Program (ESAP) has been implemented as part of the donor funded support for basic services (Khan et al., 2014), starting from 2006 and continuing to this day. ESAP is currently in its third phase and it supports interventions in the five basic service sectors in almost half of all districts in Ethiopia (https://www.vng-esap.org/). The implementing agencies that work within ESAP employ a range of accountability tools that primarily target the “citizen-service provider relationship,” but some organizations work with participatory and gender-responsive budgeting tools (Nass et al., 2018), which tackle accountability challenges encountered “higher-up” the decision making chain. In addition to ESAP, there are a range of government sponsored, sector-specific accountability initiatives throughout the country. In 2016, the Ministry of Health in Ethiopia started to implement a pledge within its first Health Sector Transformation Plan (HSTP-I; which ran from 2015/16 to 2019/20), to provide a citizen engagement opportunity for all Health Centers and Primary Hospitals. The engagement opportunity was established in the form of a “Community Score Card” intervention, and initially only implemented in Ethiopia's so-called agrarian regions; Tigray, Oromia, Amhara and SNNPR (e.g., described in Argaw et al., 2019).

A visit to Ethiopia in 2018 by the authors of the Lancet Commission report on High Quality Health Systems in the Sustainable Development Goals-era, fueled the discussion regarding Ethiopia developing a *nationwide* social accountability intervention in the health sector as a mechanism to drive quality-of-care improved health systems. As a result, H.E. Dr. Seharla Abdulahi, State Minister of Health, asked one of the lead UN agencies in Ethiopia to play a coordination role regarding the monitoring of implementation of the Community Score Card in the remaining, predominantly pastoralist, regions. This involved revising the model based on lessons learnt from the initial implementation in order to maximize the opportunity to strengthen health system quality improvements. Subsequently, tentative steps were undertaken to look into the roll-out of the Community Score Card in Ethiopia's remaining regions. The Ministry of Health received support from the UN to prepare each region for the use of citizen feedback tools. Especially in areas where citizen engagement had not yet been well established, it was accepted that several interim steps may need to be taken. This study focuses on the efforts to establish an interim accountability intervention in one of these remaining regions: Ethiopia's Somali Region.

## Background

### Health sector bottlenecks in Somali Region

Health outcomes in Ethiopia have improved significantly in the past 20 years. According to Demographic and Health Survey research, key indicators such infant- and under-five mortality rates have all improved, decreasing from 77 to 47 infant deaths per 1,000 live births and from 123 to 59 under-five deaths per 1,000 live births between 2000 and 2019 (Central Statistical Agency [Ethiopia] and ORC Macro, 2006; Ethiopian Public Health Institute, Federal Ministry of Health, The DHS Program and ICF, 2019). However, regional disparities are significant and the predominantly pastoralist areas of Afar and Somali Region often have the worst health outcomes. The 2019 DHS shows that Somali Region had the highest under-five mortality rate in the country, at 101 deaths per 1,000 live births, and the second-highest infant mortality rate, at 71 per 1,000 live births; compared to an under-five mortality rate of 26 per 1,000 live births, and an infant mortality rate of four per 1,000 live births in the capital Addis Ababa. The same report showed that antenatal care (ANC) coverage from a skilled provider was highest in Addis Ababa (97%) and lowest in Somali Region (30%) and that percentages of women using modern methods of contraceptives are lowest in Somali (3%) and Afar (13%) Regions, compared to 50% in Amhara and 48% Addis Ababa (Ethiopian Public Health Institute, Federal Ministry of Health, The DHS Program and ICF, 2019).

Ethiopia is a federal nation in which individual state presidents and their local leadership wield enormous power. With the blessing of the central authorities in Addis Ababa, state governments set the tone for the developmental agenda and regional priorities. Meles Zenawi, who was in power from the early 1990s until his death in 2012, and his successor Mengistu Haile Mariam, implemented a successful (from a health-outcomes perspective), but two-speed, developmental state agenda in Ethiopia (Fetene et al., 2016; Assefa et al., 2017; Melaku and Shi, 2017). Many national policies and strategies to improve the health and wellbeing of the nation were initially rolled-out in the more densely populated “agrarian regions” which included Amhara, Oromia, Tigray, and sometimes the Southern Nations and Nationalities Region (SNNPR), while the pastoralist-dominated regions such as Afar, Somali Region, Benishangul-Gumuz, were only able to implement these programmes and policies at a much later stage, if at all. One example of this phased approach is Ethiopia's Productive Safety Net Programme, the largest social protection programme in sub-Saharan Africa, which was launched in the agrarian regions in 2005, while pastoralist areas had to wait until 2008 to receive the same life-saving benefits (Alene et al., 2021, p. 2). In the first 10–15 years of the new millennium, health indicators in the most populous parts of Ethiopia improved dramatically, Millennium Development Goals were reached and the country was recognized for its health leadership (Fetene et al., 2016; Assefa et al., 2017). In Somali Region, improving the health of the population was less of a priority; the regional government focused instead on the suppression and containment of the secessionist Ogaden National Liberation Front (ONLF) rebels, and those suspected of being associated with them (Economist, 2019). In 2018, the 15-year reign of Somali Regional State President Abdi Mohamed Omar came to an end. The Economist called “Somali Region before August 2018 … the most ill-treated place in all of Ethiopia, tyrannized by its then state President Abdi Mohamed Omar who had waged a scorched-earth campaign against secessionist rebels for more than a decade.” According to Human Rights Watch, the heavily armed special police force in Somali Region, the Liyu, “murdered and raped civilians, imprisoned and tortured tens of thousands of alleged rebels” (Human Rights Watch, 2018). Years of anti-secessionist activity, ethnic conflict in areas where Somali Region borders other Ethiopian ethnic groups, and severe droughts, led to significant internal displacement of populations within Somali Region, which also hosts refugees from neighboring Somalia and Eritrea. In 2019, the joint Government, UN and NGO protection cluster recorded the presence of over 1 million displaced people within Somali Region, 68% of whom were displaced due to conflict (Somali Protection Cluster, 2019).

During Abdi Mohamed Omar time in office, the Somali Regional State's healthcare system did not meet the needs of all those living within its boundaries. The health outcomes in Ethiopia's Somali Region are among the worst in Ethiopia (UNICEF, 2020). Analyses from UNICEF and other agencies suggest that shortcomings in every aspect of healthcare provision hamper Somali Region's health system ability to deal with shocks: “On the supply side, health facilities have limited drugs and trained staffs. Between 2016–2019, outbreaks of measles were a huge concern, particularly in drought affected areas” (UNICEF, 2020, p. 15). A study for the UN Development Programme in Ethiopia highlights the main reasons why the impact of their governance interventions was below par in Somali Region: …“structural weakness in institutional vision, objective setting and strategic planning; lack of efficiency in resource allocations; weak system for accountability; poor access to information that in turn weakens managerial capacity for sound decisions and optimizing resource use” (UNICEF, 2020, p. 8). When this research was conducted in 2019, it was clear that the literature best suited to guide the health system financing research was that pertaining to fragile and conflict-affected states (Bertone et al., 2019; World Health Organisation, 2020).

### Promoting accountability in the health sector

**Figure 1:**
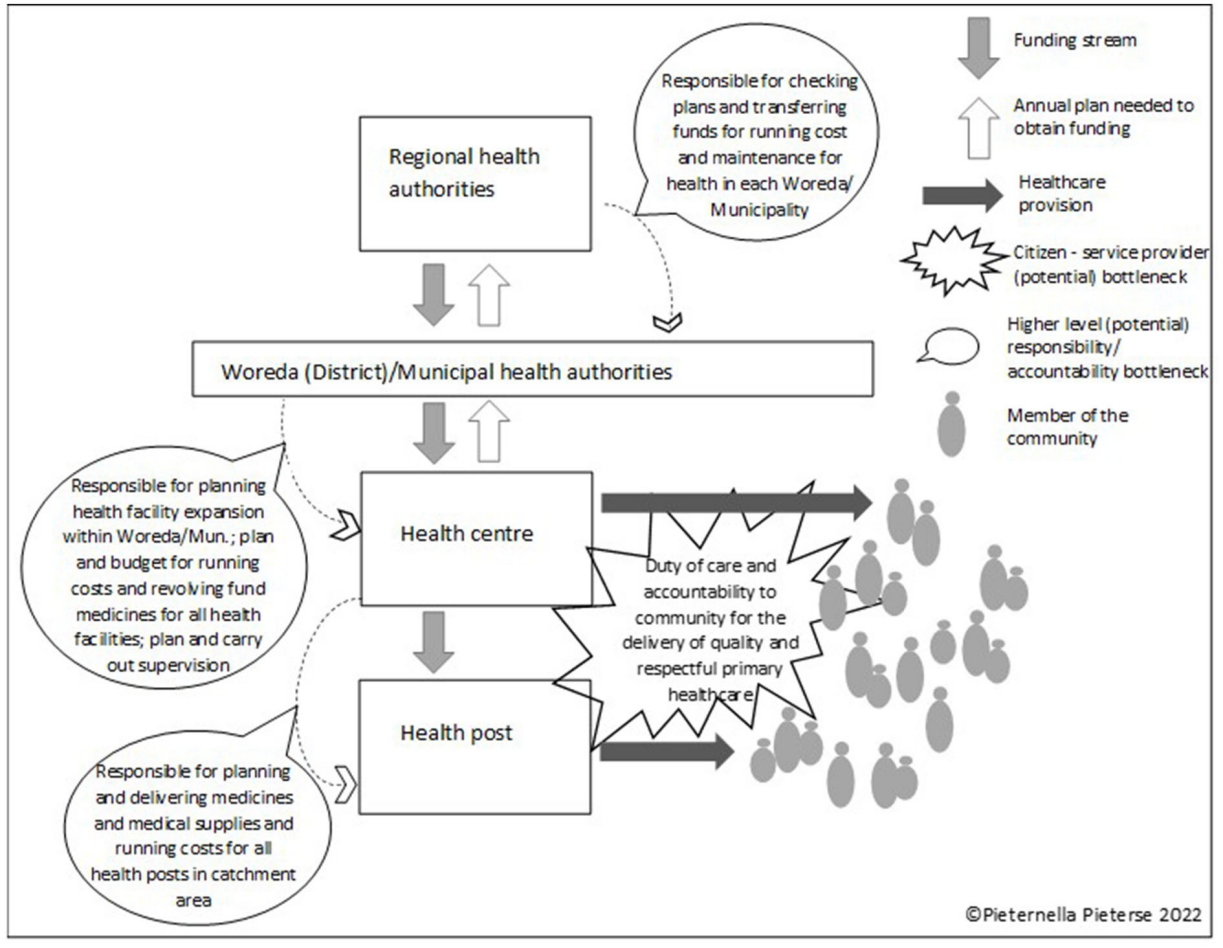
Key “bottlenecks” where a lack of accountability can undermine health service provision.

This study focuses on the efforts to establish an interim accountability intervention in Ethiopia's Somali Region. When assessing accountability relationships in the health sector in LMICs, there are a number of key “bottlenecks” where a lack of accountability can undermine service provision (see Figure 1). Accountability problems most commonly occur between patients and health service providers, whereby the latter may not listen or be disrespectful; healthcare staff may make insufficient effort to correctly diagnose a patient; a healthcare provider may not utilize the right resources to attend to a patient properly; or patients may be extorted by service providers or asked to make an unauthorized payment for medicines or medical commodities to allow health workers to replenish their stocks (e.g., Lodenstein et al., 2017). Another “bottleneck” where accountability challenges often occur is situated between local-level health authorities and health facilities, whereby budgets, supportive supervision and general support for health facilities are not extended as optimally as they should (Brinkerhoff, 2004). Issues of misallocation, suboptimal prioritization of funding or fraud can also occur at regional and national level, depending on a country's health system (Savedoff and Hussmann, 2006). Accountability challenges at each level affect one-another, with a lack of accountability at higher levels of authority (at national, regional or district-level management in charge of supervision, supplies or budgeting) severely impacting the ability of the frontline service providers to deliver health services in a satisfactory manner (Cleary et al., 2013).

### Accountability in health in Ethiopia

Ethiopia's Community Score Card intervention is an accountability intervention that encourages citizen to hold service providers accountability for the provision of decent and respectful healthcare. A citizen scorecard exercise involves the quarterly gathering of scores, or quality ratings, from a significant number of patients and community members, indicating their satisfaction with the way health services are being extended to them. The score card exercises, as they are being employed in Ethiopia, are being organized by Health Extension Workers (HEWs) who primarily provide community based health services, and by their “Health Development Armies,” groups of volunteers who support local HEWs by raising awareness of simple public health topics such as vaccinations, hygiene, good nutrition, etc. (Yitbarek et al., 2019). The feedback gathered quarterly is supposed to be discussed at health facility level where areas of improvement and improvement plans are recorded and submitted to the regional health bureaus for verification (Argaw et al., 2021). Good examples of community score card success are cases whereby health facilities have created more focus on respectful and compassionate care, while citizens' demands for running water or upgraded toilet facilities at clinics have led to budgets for such improvements being made available by local authorities (Argaw et al., 2019). The efficacy of the community scorecard programme depends on health facility staff being willing and able to respond to the suggestions of citizens in relation to the care that is being provided. Health facilities can only respond to citizens' healthcare demands, if they are in a position to provide at least a minimum standard of care, are unconstrained by budget shortages, and if they can occasionally access grants for maintenance and infrastructural improvements.

In Ethiopia, districts are known as woredas, which are the third-level administrative division of the country (after regions and zones). Liu and colleagues describe the woreda health office as the link between “national- and regional-level leadership, where policies are formulated, to the facility- and community-level, where services are delivered. As the most frontline primary care administrative body, woreda health offices are responsible for planning, resource allocation, execution, monitoring, and evaluating of primary healthcare services” (Liu et al., 2020, p. 2). The woreda health offices supervise and coordinate primary care services for catchment areas of approximately 200000 population, including oversight of 4–5 health centers, 20–30 health extension workers, and, in some cases, a primary hospital” (Liu et al., 2020, p. 2). At woreda (or municipal) level, a lack of capacity to effectively plan and budget for the required health services within the catchment area, can result in overall budget shortages, which in turn can lead to stocks-outs of medicine, a lack of funds for running costs and maintenance and health facilities, and insufficient supervision due to a lack of vehicles, fuel, etc. It is therefore logical that *higher level accountability challenges* need to be addressed first, before patient/citizen – service provider accountability can be fully addressed. This does not suggest that citizen-service provider accountability is less important, however, evidence suggest that when a health facility lacks staff and basic commodities, healthcare uptake is likely to be reduced (and all health outcome data on Somali Regions suggests this), it is therefore better to tackle this bottleneck first, and citizen-service provider accountability later.

The author was able to witness firsthand how an otherwise successful citizen-service provider accountability intervention was implemented in a setting whereby little of no woreda health authority-healthcare provider accountability existed: an ESAP citizen engagement intervention in the Afar Region's health sector. Afar Region, like Somali Region, is dominated by pastoralist communities. Despite successful “citizen - health service provider” dialogue in the Afar intervention, progress had been hampered by the lack of engagement of the woreda health office. The Afar-based health facility staff engaged in the accountability interventions were unable to access *additional funds* to respond the citizens' demands (which had included a request for more medicines and running water at the health facility). In addition, some of the managers of health facilities stated that they did not receive a budget for running costs from the woreda health office. It was evident from this accountability intervention, that it is impossible for healthcare workers to be responsive to the needs of citizens, when they are unable to access sufficient funds to provide basic healthcare due to an accountability bottleneck at a higher level.

This lesson shaped the focus of the Somali Region rapid research: A working hypothesis was further developed by conducting a series of key informant interviews in Addis Ababa with UN and donor agency staff with experience of working in the health sector in Ethiopia; a review of a very limited amount of available literature on health governance in Somali Region (e.g., Sharma et al., 2015; Zepro and Ahmed, 2016; Usman et al., 2019; UNICEF, 2020); and key informant interviews in Jigjiga, the capital of Somali region. Many sources indicated that it is likely that potential healthcare inefficiencies in Somali Region are caused by a lack of capacity for sound healthcare budget planning and budget execution at woreda-level, which thus became the most important area of accountability to focus on.

### Identifying a suitable interim accountability intervention

The UN agency commissioning this research aimed to fulfill their mandate of supporting *citizen engagement in the health sector* Somali Region at the earliest opportunity. The objectives of the research were therefore to:

- demonstrate that this may not yet be the right time to deploy the Community Score Card, by showing that higher level accountability bottlenecks exist that may need to be prioritized.
- suggest other forms of citizen engagement that can improve accountability in the health sector and improve Somali Region's readiness for the extension of Ethiopia's Community Score Card.

The citizen engagement intervention that was proposed to address accountability challenges at healthcare budget planning and budget execution-level in Somali Region, was called a “citizen audit.” Citizen audits have also been called social audits, participatory audits, community audits or social accountability monitoring (e.g., Mugizi, 2013; Guerzovich et al., 2017). A citizen audit is an intervention whereby a select group of citizens are assisted by NGO experts to conduct an audit of the budget plans and budget execution of an entity of concern. Once citizens have gathered their evidence, which can be simple proof of stock-outs in a local health facility, or a lack of funds to repair an ambulance, a dialogue is entered into with the budget making and budget executing authorities to discuss the findings and to come to an agreement on how future budgets can be improved on, in order to benefit a wider population (Guerzovich et al., 2017). In principle, many different types of expenditure can be audited, though the most common form focus on a budget, or a dedicated section of a budget at national, regional or local level. The citizen audit approach, like most other social accountability tools, is adaptable to the context in which it will be applied. Citizen audits bring budget makers closer to the end users of the services that they make plans and budget for, which is designed to make them focus more on optimizing budgets for maximum citizen utility. Citizens engaged in citizen audits learn to constructively engage with budget makers, and do so at the right time within the budget cycle. The specific design for the Somali Region involved the engagement of technical staff of the Regional Health Bureau, who appeared to be unaware of the capacity gap they are dealing with at woreda-level, or unable to raise this issue with the relevant authorities who could take action to improve capacity support. A UN-funded, NGO-supported citizen audit, in advance of a roll out of the community score card, could potentially uncover a lack of technical capacity at woreda level and a lack of guidance and support for budget planning and execution at the woreda health offices in Somali Region. This, in turn, could lead to greater support and technocratic engagement from UN experts and from staff at the Regional Health Bureau, to ensure that woreda-level health budgets were optimized for best health outcomes throughout Somali Region. It was envisaged that regular future citizen engagement opportunities at budget level would become an opening to create more demand-driven health services.

### The funding flow of Ethiopia's health budget

The large majority of funding that comprises the health budget in Ethiopia covers health worker salaries, which are not influenced by woreda-level budget making. The budget that woreda health officials have influence over covers the running of the health bureau itself, health bureau costs for the supervision of health facilities and the transportation of medicines and other commodities, the running cost budgets for health facilities, and small capital investments for the maintenance of health facilities (UNICEF, 2017). Ethiopia's federal system ensures that funding for public services is equitably disbursed from the national to the regional governments, based on population size. However, the relationship between the Addis Ababa government and Somali Region had been strained before the 2018 change of leadership, which may have affected staffing and budget transfers, impacting healthcare provision in Somali Region (Carruth, 2016). The Ethiopian government's effort to decentralize health care delivery in the past 15–20 years (Federal Democratic Republic of Ethiopia, 2011; UNICEF, 2014), has meant that the Somali regional government has slowly been given more autonomy, and that capacity for equitable health budget decision making is only now coming into focus.

## Materials and methods

The research questions posed by the UN consultancy were as follows: (1) is Somali Region ready for the roll-out of Ethiopia's Health Community Score Card, and (2) if not, what interim intervention can be implemented to prepare for the roll-out of Ethiopia's Health Community Score Card?

In order to answer these questions, the researcher had to choose a research methodology that took account of the opportunities and constraints that presented itself before and during the research. Time and budget were major constraints, which is why a *rapid research* method was chosen. Preparation, field research and analysis had to be completed within one month and there was no budget for research assistants. The researcher was accompanied during the field work in Somali Region by a programme manager of the commissioning UN agency and had use of a UN vehicle with driver.

### Research methods

For this rapid research, the onus was on demonstrating that the budget planning processes in selected woredas in Somali Region were of varying quality, thus depriving some health facilities of access to sufficient running cost funding to adequately operate, and making it unlikely that health facilities would be able to access additional woreda-level funds to respond to citizens' demands for health service improvements. Suboptimal healthcare delivery was expected to be found in locations where woreda health office lacked planning and budgeting capacity, and lacked support from technocrats and/or the regional health authorities.

To carry out this research, the normal case study research methodology (Yin, 1994, 2009; Tellis, 1997) was condensed to create a rapid case study approach, whereby the focus was on creating sufficient evidence to justify designing an intervention, which itself would yield further evidence of possible healthcare budgeting capacity constraints. The rapid case study approach aimed to produce a series of “impressions” of the link between the woreda head of the health office's budgeting capacity and the functionality of at least one health center and an associated health post (these are small primary healthcare facilities usually staffed by two Health Extension Workers, and fall within the management structures of health centers) within the same woreda.

The case study method was chosen as it accommodates

“…the fact that the context contains innumerable variables-therefore leading to the following technical definition of case studies: [Case studies are] research situations where the number of variables of interest far outstrips the number of datapoints (Yin, 1994, p. 13).”

These “impressions” were primarily shaped by interviews with key informants, budget details provided by all, as well as observations made at the woreda health offices, the health center and health post facilities. Key informants included: (i) heads of woreda health offices, (ii) heads of health centers in the same woreda, and (iii) heads of health posts that are within the management of the health centers that were focused on. The key informant interviews used semi-structured guidelines that followed the assessment framework (Table 1). For the woreda health office heads, questions focused on (i) budget planning processes, (ii) the evidence base for the annual budget that is being used by the woreda health bureau heads, as well as (iii) the guidance for budget making received from the Regional Health Bureau, (iv) consultation processes employed to elicit suggestions from the hospital and health center leadership, and/or (v) any other idiosyncrasies that can be noted regarding the woreda-level budget planning process. For those in charge of the health centers and health posts, the interviews focused on (i) the operating budgets from their facilities, (ii) budget shortages, (iii) stock-outs, and (iv) the opportunities for engaging in the planning and budgeting process at woreda level. Interviews were conducted in English when interviewees indicated being comfortable with the language, and in Somali otherwise. During Somali-language interviews, the accompanying UN programme staff member translated the questions and answers into English. The interviews were not audio recorded; extensive notes were taken during all interviews and these were transcribed to create short overviews of each conversation (*n* = 18).

**Table 1:** Framework of assessment and analysis to establish healthcare delivery bottlenecks at woreda/municipal level in Ethiopia's Somali Region.

**Area of focus**	**Who to focus on**	**How to assess**
Planning capacity:		
• Elaboration of planning process • Budget engagement from Regional Health Authorities • Population data available (up to date?) • Health facility engagement • Awareness of number of health facilities and their needs • Reliance on previous years' plans • Strategy to update previous years' plans • Evidence of evaluation of execution of previous years' plans • Evidence-based planning (which evidence?)	• Woreda/municipal Health of Health • Health Center Director/Manager • Health Post In-Charge	• Interview • Available documentation • Participant observation: – Woreda: availability of paper documentation, computer, laptop, electricity, internet connection – Health facility: visible patient activity, medical supplies available in store (in/out of date?), electricity, running water, administrative office (computer/laptop, internet connection)
Budgeting/ budget execution (woreda/municipality):		
• Health facility engagement • Management of cost-recovery medicine fund • Engagement with health facilities re budget shortages • Allocation of discretionary funding (block grant) to woreda priorities vs. health facility priorities? • Woreda/municipality on track re: funding disbursements, according to budget cycle • Under/overspending (if so, why?)	• Woreda/municipal Head of Health	• Interview • Available documentation • Participant observation (incl. availability of computer, laptop, electricity, internet connection)
Budgeting/ budget execution (health facility):		
• Budget engagement with woreda/municipality • Allocation of cost-recovery medicine and management of ‘recovered' funds? • Engagement with woreda/ municipality re budget shortages (are there shortages?) • Amount of running cost allocation and prompt transfer of funds? • Other funding sources (if yes, pls. elaborate)	• Health Center Director/manager • Health Post in-charge	• Interview • Available documentation • Participant observation at health facility/ health post: visible patient activity, medical supplies available in store (in/out of date?), electricity, running water, administrative office (computer/laptop, internet connection)
Oversight:		
• Budget oversight plans • M&E visits planned, budgeted and executed	• Woreda/municipal Head of Health • Health Center Director/manager	• Interview • Available documentation
Implementation (woreda/municipality):		
• Self-assessment of current successes and challenges at woreda and health facility level	• Woreda/municipal Head of Health	• Interview • Available documentation
Implementation (health facility):		
• Self-assessment of current successes and challenges health facility level • Stock-outs of essential medicines • Staffing • Availability of ambulance • Outreach visit for vaccinations • Ability to support and supply Health Posts	• Health Center Director/Manager	• Interview • Available documentation • Visual inspection of health facility pharmacy

The second method of data collection was observation; which is about exploring people's actions and behavior (Patton, 2014) as well as examining objects, occurrences, events and interactions (Gill and Johnson, 1991). The main areas of observation were the locations where the interviews took place; the woreda health offices, the health centers and the health posts, and the level of equipment and readiness for service that was evident during the interviews. The researcher asked all woreda heads of health to show her the annual plan and budget to observe how easy it was for them to access this data. This question usually demonstrated whether the office computers were functional and whether there was electricity. In health centers, the researcher asked to be shown the medical supplies in the pharmacy, and in health posts she asked to view the medical supplies cupboard/ storage area. Observations were described during or after each interview and added to the interview descriptions. For each of the research locations, bullet-points of the main findings on the woreda/municipal head of the Health Office were placed in table beside the findings on the health facilities, deliberately arranging these data points to show the connections between the two (see Table 2, case studies 2.1–2.5 in the Results section).

**Table 2:** Short case studies based on Woreda/Municipal Health Office - Health Facility visits.

**Case 2.1: Good municipal leadership, well-funded, well-run Health Center (HC)**	
**Municipal Health Office**	**Health Center**
• Budget for primary healthcare provided by municipality. • Experienced and committed municipal health leadership, able to attract regional resources for additional construction, clear planning and budgeting, accountability structures in place.	• Well-funded and well-functioning HC, if anything slightly overstaffed, but HC on course to become primary hospital. Located beside nursing college, which makes it a good choice for upgrading to hospital.
• Running costs as per budget (not disclosed)	• Running costs received exactly as per budget, no shortcomings
**Case 2.2: Inexperienced municipal leadership, under-resourced and unsupported HC**	
**Municipal Health Office**	**Health Center**
• Inexperienced Head of MHO, located in town, away from one HC in municipality. • Head has hired unsuitable MHO staff, wasting resources. • Plans to construct office for municipal health (self), more waste of resources. • Does not seem to allocate any running costs to HC, does not know that HC uses alternative health financing arrangements. • Impossible to tell what will happen with capital + block grant funding allocated to running of MHO.	• Previously underperforming HC boasted by hiring of two medical doctors financed by the municipal mayor. • HC uses revolving medicine funds to run its affairs, it should receive greater budget drug allocation to make best use of funding flowing to MHO. • Because this is the only HC in municipality, funds allocated to MHO should be devoted to further upgrading of HC buildings and completion and stocking of HP under construction.
• Running costs per HC: 10,000 ETB/month	• Received: none (using health financing instead)
**Case 2.3: Uninformed Woreda Health Office leadership, underfunded HC**	
**Woreda Health Office (WHO)**	**Health Center**
• Head of WHO seems focused on construction of new Health Posts in woreda, less focused on supporting existing HC and Health Post (HP). • Head of WHO seemed unaware of running costs going to HCs. • WHO is unwilling to pay salary supplements to medical doctors entitles to hardship allowances. • WHO denied there were any problems with transfer of running costs, despite several heads of HCs complaining about this and recounting recent meetings about it with WHO head.	• Decent HC leadership constrained by lack of funding which seems to stem from inability of WHO to transfer running costs as budgeted. • HC had recruited a medical doctor after 6 months of searching, but he stayed only for 2 weeks and resigned when he realized that duty payment and other hardship allowances would not be paid.
• Running costs per HC: 20,000 ETB/month	• Running costs received: None for 8 months, 1 ×7,000 ETB recently
**Case 2.4: Decent Woreda Health Office leadership, well-run HC**	
**Woreda Health Office**	**Health Center**
• Well run woreda health office with well qualified staff. • WHO staff complains of not receiving sufficient running costs for its main Health Center or running of WHO. • WHO successful in accessing capital funds for the construction of a new HC and two new HPs.	• Seemingly well-run HC that is expanding to accommodate operating theater and emergency obstetric surgery room, in process of recruiting additional medical doctor and surgeon. • Appears to have diaspora donors. Running costs low but consistently paid by WHO and HC appears able to run on this tight budget. • Staff expressed desire to receive additional running costs to expand vaccination coverage and improve services further.
• Running costs per HC: 12,000 ETB/month	• Running costs received: 12,000 ETB/month
**Case 2.5: High turnover of Woreda Health Office leadership, minimal resources for health facilities**	
**Woreda Health Office**	**Health Center**
• Head of WHO has only been in job for 2 months, and the 3 people in the post before him lasted no more than 2–4 months each, reflecting the political turmoil in Somali Region that has affected some areas more than others. • Head of WHO was not around during budget negotiations, and clearly if nobody defends the health sector budget, the allocation will be minimal. • The WHO head is supposed to manage 5 HCs but admits there are barely any funds to run the office, allocate drugs to all HC and HP, manage the allocation and distribution of free medication and carry our supervision. • WHO head seemed daunted by challenges and expectations his job provides. • WHO head locked out of office, watchman missing, does not seem to work from office, no electricity at office, no computer/laptop.	• HC that seems well run but completely constrained by a total lack of budget, medication, support, everything. Remote location. • The HC has adopted survival strategy of using its income to run its services, which does not seem to have been sanctioned by relevant authorities - but it has been sanctioned by the hospital board. • It is clear that the HC could do so much better if there were more funding available. • Ambulance unavailable; no funds for repairs. • Minimal budget for cost recover medication. • No funding for routine maintenance or for support to HPs. • The catchment communities lack basic service provision, as HPs have no medication and are barely able to provide vaccinations.
• Running costs per HC: 10,000 ETB/month	• Running costs received: none

### Sampling

Due to aforementioned time constraints, a total of five research locations were selected within Somali Region, with the objective of gathering data from the greatest possible diversity of settings. Two of the study locations were municipalities—where the health offices were in charge of the budgeting for fewer, but busier, hospitals, that served urban populations and served as a referral hospital for smaller facilities nearby. The other three were rural woreda locations—which each managed the budgets for several health centers per woreda and a multitude of health posts that were managed, from a budgetary perspective, by the health centers. The UN agency that commissioned the research supported the selection process of the woredas, to include a mix of remote locations, areas closer to the regional headquarter town of Jigjiga, and a thriving border town close to Somalia. The study location selection was influenced by the need to avoid areas deemed unsafe to travel to, due to ongoing conflict or insecurity.

## Framework of assessment and analysis

Since 2008, the Organization for Economic Co-operation and Development has compiled a list of “countries and contexts” that have been considered fragile or conflict affected, and Ethiopia is one of 27 countries that have appeared on this list every year, and is therefore considered chronically fragile (Organisation for Economic Co-operation and Development, 2018, p. 27). According to the World Health Organization (WHO) “…countries considered as fragile or affected by conflict, have significantly higher out of pocket expenditure, external dependency and health related impoverishment. They also have lower mean government expenditure on health in relation to wider government expenditure and total health expenditure” (2020, p. viii). Ethiopia's Somali Region fits the WHO descriptions of a fragile and conflict affected area. For this reason, little health expenditure data was collected, as the total real health expenditure per person, was most likely an unpredictable sum of government, UN/aid donor, NGO and out of pocket expenditure. It was also unclear as to which types of data could be collected, how accurate budget data might be and how this could be analyzed and compared across locations, given that there was no recent census data for Somali Region in 2019. Whilst some budget data was collected at each of the five study locations, variables such as population, number of facilities, etc. made it impossible to compare like with like, therefore, more emphasis was placed on the amounts of funding that facilities had received for running costs and “revolving fund” medication, as planned and actual expenditure can vary considerably (Piatti et al., 2022).

To create a suitable framework of assessment and analysis for the examination of woreda and municipal health planning and budgeting capacity, a range of literature was consulted. Based on the available literature, the following four headings were selected (see Table 1): Planning capacity; budgeting/budget execution; oversight; implementation. Some sub-headings were added by the author based on Ethiopia-specific experience and literature (Fetene et al., 2016, 2019; Liu et al., 2020).

The following literature was used to construct the assessment framework:

- Health systems analysis, health systems financing assessments: (USAID, 2012; Asante et al., 2016; Kumah et al., 2020; Hanson et al., 2022).
- Fragile and conflict affected states healthcare literature: (Health Systems Global, 2016; Bertone et al., 2019; Dong, 2019; Jowett et al., 2020; Witter et al., 2020; World Health Organisation, 2020).
- Assessing decentralized management/budgeting capacity in LMICs, FCAS: (Newbrander et al., 2012; Daire et al., 2014; Barasa et al., 2017).

## Results

A total of 18 interviews were conducted, the Regional Health Bureau-Head of Planning, Budgeting, Monitoring and Evaluation, NGO Coordination & Resource Mobilization, was interviewed twice, at both the beginning and end of the research visit. In all five study locations the head of the woreda health office was interviewed as well as the head of one hospital or health center that fell within the catchment area of the same woreda. In three locations the head of a health post was also interviewed. The inclusion of 2–3 interviews per study location allowed for the creation of rough “impressions” that illustrated the relationship between the apparent competence and attitude of the woreda health office head and the situation at the hospital, health center or health post. Table 2 shows five case studies created by triangulating the interview data and observations of the heads of health offices and corresponding directors of health facilities.

### Woreda health office capacity

In two cases, serious, capable and well-informed woreda health office heads talked at length about the challenges of their jobs and their struggles to do more for the health centers; it was obvious that the health facilities they supported reaped the benefit of their commitment. Several woreda head of health offices tried to invest in the construction of additional health posts to serve remote communities, and were in the process of securing staff training for suitable individuals from nearby communities.

All five heads of woreda health offices faced many challenges, the budgets they managed were small and there were a lot of demands. One woreda health office head was relatively new in the post and his department was, by his admission, significantly underfunded. The health center nearby struggled to cope with financial hardship. In most cases, the interviewees appeared honest and no information that was provided at the woreda health office was contradicted by facts found at health center and health post levels. There was one exception; one office head and the finance officer provided lengthy responses to questions about their work, the health facilities, their ambition to create more health posts. There were many contradictions and a reluctance to share data; the nearby health center struggled with finances and the health center's director admitted not receiving the budgeted running costs for months, and having to follow up with the health office about the payment of overtime and hardship post allowances on behalf of the staff. All woreda heads of health offices agreed that there were few guidelines that they had to adhere to when creating their annual health budget.

### Running costs

The set rate of monthly payment for a health center's running cost, and whether it was paid regularly, became a key indicator, which was added to the short case studies (Table 2). At the four more remote locations (which included one small municipal health center) running costs budgets of between 10,000 and 20,000 Ethiopian Birr (€293–€587) per month were encountered, the large municipal hospital, understandably, had a much larger budget and was therefore not taken into account. Running costs for health centers also cover expenses for the running of health posts under their supervision. One health center received the exact budgeted amount every month; two health centers reported receiving no running cost finances from the woreda health offices, despite a running costs budgets being agreed, and the fourth had received no funding for 8 months, followed by a one-off disbursement the previous month of half the budgeted amount. Most health facilities reported using the money they received from the sale of medicines to cover their running costs, which was manageable in the larger health centers, but much more of a challenge in health posts that served remote poor populations. The income from charging patients for so-called “cost recovery medicines” was supposed to be transferred to the regional health office, which uses the recovered funds to buy new stock on a quarterly basis *via* the regional health bureau, but many health facilities had come to an agreement with regional authorities that the funds could be kept at the facility and used to cover running costs.

### Observations on woreda health office locations

Observations played a much larger role in the sketching of the five “woreda health office - heath center” relationship case studies that initially anticipated. The locations and quality of the woreda health offices seemed to tell their own stories, especially when office spaces were rented, which demonstrated a particular budget decision. Observations regarding electricity availability and the presence of functioning desktop computers or laptops were also telling, as they suggested how well computerized tasks such as budget management could potentially be carried out. Three woreda health office heads were interviewed in government-owned offices close to the health facilities or in a cluster of government buildings. In certain locations in Somali Region where population growth has been significant, government-owned offices do not exist and renting office space is fully justifiable. The choice of rented office accommodation was illuminating: One woreda health office head met us outside his locked-up woreda health office where, on a work day; he was unable to enter the building as the watchman had disappeared with the keys. The rented building, a large western-style three story residence at the edge of the town, had several indoor bathrooms but no running water, and no electricity due to a problem with the generator. Every office chair on the semi-furnished second floor was slightly broken, and there was no computer in sight. The head of the health office explained he had only been in the job for 2 months, and that none of his predecessors had lasted more than 2–4 months in the job, due to political upheaval and an apparent lack of interest in taking on the responsibilities of the post.

At a fifth location, the municipal health office was also located in a rented space. It was some distance from the large health center where, it turned out, the official municipal health office space was still available. The rented office was a windowless room opening out onto a bustling shopping street in the busy border town. There was electricity, but the computer that sat on the desk was “out of order.” The municipal health office head showed the annual budget on his mobile phone and introduced us to a group of young women who were “health education officers,” hired directly by him, instead being based out of the health facility, which would be more common practice. Most of the annual health budget, for the coming year, this head of office explained, was going to be dedicated to constructing a new building to house the health office.

### Health centers

The variance of woreda health office head capacity, their interest in and priorities for their health budgets appeared to have a significant impact on the running of health facilities. Those that were almost entirely dependent on woreda/municipal funding appeared to suffer most from a lack of resources and a lack of effort from the woreda/municipality to create access to discretionary funds that should be available at woreda, municipal or regional level. One hospital director explained that he had spent 6 months recruiting a medical doctor, who resigned after 2 weeks, when he realized that duty payment and other hardship allowances would not be paid (these payments were at the discretion of the woreda health office). Another hospital director explained how there was no budget to repair the vehicle the facility had, leaving the health center without an ambulance or opportunities to carry out vaccination visits to remote locations. While the use of the cost-recovery medicine income was sanctioned by the hospital board, it seemed to have created friction at the woreda health office level. Unfortunately, the health center director said, “we have no other option” [interview 6].

### Health posts

The health posts, small primary care facilities, which are managed by nearby health centers, appeared to suffer most from the lack of financial support that should flow from the woreda to the health center and onwards to each of the health posts. Because health centers had insufficient running costs, they often lacked fuel to carry out visit to health posts for supervision and for the delivery of medication. It also impacted the number of outreach visits medical teams could carry out to provide immunizations for children under five in remote locations. In one location, a Health Extension Worker (HEW), the sole trained staff member in charge of the health post, explained that the delivery bed in her small clinic had been broken for over 6 months. The lack of funds to repair it forced her and the local traditional birth attendant to deliver babies in people's homes instead of the health post. The HEW spoke about the fact that regular funding could create the opportunity to have no more home deliveries in her locality, if only she could afford to repair the delivery bed. She added that funds for a recovery bed would allow her keep mothers and newborn babies at the health post for observation, which she should do according to WHO clinical practice guidelines. In another health post, the medic in charge, a trained nurse, explained that his health post relied on an international NGO to regularly drop off free medicines such as anti-malarial medication, as the health center or woreda do not have fuel to deliver these supplies. He travels to the nearest town to collect his wages every month and spends a small portion of his own funds to buy medical gloves, as he never receives enough of them.

### Coping strategies

All health facilities appeared to have a range of mechanisms to cope with shortages and to deliver the best possible care they could manage. In one of the wealthier localities, the mayor of the town had stepped in and provided funding for the salaries of two medical doctors. At a different location, a diaspora donor, a Somali region citizen living abroad, had funded a surgical theater for emergency obstetric care. As mentioned above, in almost all locations the “cost-recovery” medication income was used to cover running costs. Links with international NGOs and UN agencies further plugged service gaps, often in relation to provision and the transportation of free medicines—which were technically only delivered to the woreda health offices for onward distribution.

## Discussion and conclusions

The rapid research was able to produce five case studies, based on the impressions gleaned from observations and interviews with heads of woreda health offices, directors of health facilities and heads of health posts within the same woreda or municipality. As stated in the introduction, the rapid research was not designed to come to firm conclusions about the challenges related to woreda and municipal-level planning and budgeting, and how this impacted on the healthcare delivery in Somali Region; the research sought to deliver five “robust enough” impressions to argue that a “citizen audit intervention” would be a suitable next step for the organization that commissioned the research.

Despite the perhaps less-than-rigorous *rapid case study approach* taken to collect, analyse and present data, the field visit yielded some important insights that had not previously been described in the literature on health systems in the Somali Region of Ethiopia. The findings that have emerged from the rapid research were similar, in some respects, to the conclusions drawn from research into woreda-level healthcare planning and management capacity, conducted in other regions of Ethiopia (Fetene et al., 2016, p. 15–16), which noted:

Higher-performing woredas had greater use of data informed problem solving, more respectful and supportive relationships with the community, and stronger support from zonal and regional health bureaus in terms of perceived transparent communication, financial support, and technical inputs. Although much of the previous literature on primary health care improvement has focused on technical inputs as paramount to building primary care systems, our work suggests that more fundamental management and governance capacity is paramount to achieving top performance.

This study adds yet another data point to a small but growing body of literature that draws attention to the need to strengthen management, planning and budgeting capacity at district level in order to improve primary health outcomes (Seims et al., 2012; Edwards et al., 2015; Fetene et al., 2016, 2019) even, and perhaps especially, in fragile and conflict affected settings.

The research report that resulted from the rapid research (presented to the agency that commissioned the research) clearly transcended the hitherto anecdotal evidence that woreda-level health budget planning remains an area that is fraught with significant shortcomings. The agency agreed that this issue would be worth focusing on for the implementation of the citizen audit intervention. Furthermore, the research provided an evidence base for the delay of the roll-out of mandated Community Scorecard implementation in Somali Region. In a context whereby health facilities remain under-resourced due to budgeting capacity constraints, a citizen - service provider-focused accountability intervention would probably have been of limited utility.

### Support for woreda heads of health offices

The evident lack of guidance that was experienced by the woreda heads of health offices was highlighted in the research report as one area that the UN agency could act upon in the short term. It was clear that some newly appointed woreda office heads has little or no relevant experience, yet they found themselves in positions whereby they needed to lead the planning of the woreda or municipal health budget. A series of formulae handed down from the regional health bureau to aid the budget planning, including prescriptive guidelines for the ordering of cost-recovery medicines, created using simple population-based calculations, could support more equitable woreda health budget making. Every woreda should be given a framework of budgetary guidelines, and each woreda should receive robust mentoring support, as described by Liu et al., who demonstrated that “a combination of intensive mentorship and structured team-based education” was successful in improving the management capacity and primary healthcare system performance at woreda-level in Amhara, Oromia, Tigray and SNNP Regions (2020, p. 5–7).

### Gender balance

While the assessment framework did not contain an indicator in relation to gender balance, the lack of women in leadership positions nevertheless stood out. All heads of woreda health bureaus, and all directors of health centers were male, while only one female health extension worker was encountered among the list of interviewees for this research, along with one female women's group representative. In total, <10% of individuals in leadership positions in Somali Region are thought to be women (UNICEF, 2020). The predominance of men in leadership positions in health, as well as other public sectors, is common in LMICs, and yet this is rarely acknowledged as an issue that should be addressed (Muraya et al., 2019). The predominance of men in decision making positions is especially problematic when public health planning and budgeting is conducted by men who are community leaders, not health professionals. Primary healthcare services are predominantly used by women and children, and the absence of their voices in the planning process often leads to services that are not sufficiently targeted to their needs. The existence of women's groups in certain locations where interviews were held demonstrate that some means of amplifying women's voices are now emerging in Somali Region, which are important for a possible next phase of developing space for greater citizen engagement in health.

### Limitations

The use of the rapid case study approach was useful in the described context because the evidence created demonstrated what was previously only anecdotally known: the fact that significant capacity constraints are a challenge in a number, but not all, of the woreda health office locations under study. Time allocated for this research was limited, but by creating mini case studies and grouping the finding “by case” to demonstrate the differences and commonalities between them, it made the cases as convincing as possible. Explicitly adding observations as a data collection method proved to be useful, quick and cost effective. Knowing what signs to look out for, and using relatively easy indicators (such as access to electricity and working IT equipment in this case), helped to add data points in each case. Creating mini case studies allowed the connection between the within-case data points speak for themselves. The presentation of the mini cases, ensured that the ‘heterogeneity of budget making capacities' across locations was revealed, which was *convincing enough* to allow for the introduction of a follow-up intervention.

However, it has to be noted that a more rigorous and more extensive study of woreda and municipal level capacity for healthcare planning and budgeting in Somali Region would fill the existing knowledge gap in relation to primary healthcare delivery in a much more convincing manner. Almost all aspects of health service delivery; planning, management and frontline services, remain significantly under-research in Ethiopia's predominantly pastoralist regions. This study has demonstrated that suboptimal performance of woreda management in the health sector occurs in Somali Region, much like it occurs in other parts of Ethiopia (Fetene et al., 2016). It is important that support should be directed toward all woredas in Ethiopia where health outcomes are noted to be below average, regardless of where in the country the under-performing area is located.

## Data availability statement

The raw data supporting the conclusions of this article will be made available by the authors, without undue reservation.

## Ethics statement

Ethical approval for this study on human participants was granted by the Somali Regional Government's Regional Health Bureau Office for Planning, Budgeting, Monitoring and Evaluation, NGO Coordination and Resource Mobilization in accordance with the local legislation and institutional requirements. Verbal informed consent for participation was obtained for this study in accordance with institutional requirements.

## Author contributions

The author confirms being the sole contributor of this work and has approved it for publication.

## Funding

The research described in this article was funded by a UN agency in Ethiopia. The assignment required the author to carry out the research, and produce the design of the intervention described in the text.

## Conflict of interest

The author declares that the research was conducted in the absence of any commercial or financial relationships that could be construed as a potential conflict of interest.

## Publisher's note

All claims expressed in this article are solely those of the authors and do not necessarily represent those of their affiliated organizations, or those of the publisher, the editors and the reviewers. Any product that may be evaluated in this article, or claim that may be made by its manufacturer, is not guaranteed or endorsed by the publisher.
